# Psychosocial correlates of depression and anxiety among treatment-seeking individuals with opioid dependence: a cross-sectional study

**DOI:** 10.3389/fpsyt.2025.1708666

**Published:** 2025-11-05

**Authors:** Siddharth Sarkar, Muzafar Pandit, Parvender Singh Negi, Rahul Mathur, Yatan Pal Singh Balhara

**Affiliations:** ^1^ National Drug Dependence Treatment Centre, All India Institute of Medical Sciences, New Delhi, India; ^2^ Department of Psychiatry, All India Institute of Medical Sciences, CAPFIMS Campus, New Delhi, India

**Keywords:** opioid dependence, depression, anxiety, quality of life, disability

## Abstract

**Background and aims:**

Opioid dependence is commonly comorbid with depression and anxiety, which contribute to greater disability and poorer quality of life. This study assessed the prevalence of these comorbidities and their socio-demographic and clinical correlates in treatment-seeking individuals with opioid dependence in India.

**Methodology:**

We conducted a cross-sectional study at a tertiary care center in North India. A total of 255 adult patients diagnosed with opioid dependence (ICD-11) were recruited after fulfilling inclusion and exclusion criteria. Depression and anxiety were assessed using the Patient Health Questionnaire-9 (PHQ-9) and the Generalized Anxiety Disorder-7 (GAD-7). Quality of life and disability were measured using the WHOQOL-BREF and WHODAS 2.0. Logistic regression was performed to examine predictors of moderate-to-severe depression (PHQ-9 ≥10) and anxiety (GAD-7 ≥10). Ethical approval was obtained from the Institutional Ethics Committee.

**Results:**

The participants had a median age of 28 (24-32) years; heroin was the predominant opioid (87%), and 30% reported injecting use. More than half (57%) had moderate-to-severe depression and 42% had moderate-to-severe anxiety, which were strongly correlated (r = 0.90). Both conditions were associated with higher disability and poorer quality of life across all domains. In univariate analyses, unemployment, stigma, interpersonal problems, and lack of abstinence were consistently linked with worse outcomes. Multivariable models showed that depression was independently predicted by stigma (OR = 2.95), interpersonal problems (OR = 4.21), and absence of opioid agonist treatment (OR = 0.30, protective). For anxiety, interpersonal problems were the strongest predictor (OR = 4.98), while past-month abstinence was protective (OR = 0.45).

**Conclusion:**

Our findings highlight the significant burden of depression and anxiety among individuals with opioid dependence, with significant implications for disability and quality of life. Future research should focus on stigma reduction, optimization of opioid agonist treatment, and integration of mental health care within addiction services.

## Introduction

Opioid dependence remains a significant global public health challenge. According to the Global Burden of Disease estimates, approximately 40.5 million individuals were opioid-dependent in 2017, and over one hundred thousand deaths were attributed to opioid overdose (1). In India, the national survey on substance use estimated that around 2.5 million people were opioid-dependent (2). Despite the availability of effective pharmacological treatments for opioid use disorders, including agonist and antagonist therapies (3), individuals with opioid dependence often suffer from co-occurring psychological symptoms that are frequently underrecognized (4).

Opioid dependence is a major global health problem that contributes significantly to morbidity, disability, and premature mortality, with comorbid psychiatric conditions further compounding this burden. Among these, depression and anxiety are especially prevalent, with international reviews suggesting that nearly one-third of individuals with opioid use disorder experience these disorders, rates far higher than in the general population (5–7). Such comorbidities not only worsen the clinical course of opioid dependence, increasing risks of relapse, overdose, and suicide, but also have a profound impact on functioning, quality of life, and social integration (8, 9). In low- and middle-income countries like India, where stigma along with various socio-cultural factors, and limited treatment access remain pressing challenges, the interplay between psychiatric symptoms, social disadvantage, and substance use may be especially detrimental (10, 11). Quality of life and disability related issues are crucial in substance use research since they reflect how people actually experience their day-to-day lives; beyond just the number of symptoms they have.

Despite existing research on comorbid depression and anxiety in opioid dependence, there remains dearth of evidence from India that blends these psychiatric symptoms with functional outcomes such as disability and quality of life beside contextual factors like stigma, interpersonal difficulties, and treatment status. This gap highlights the need for multidimensional assessment within treatment-seeking populations, especially in low- and middle-income settings where psychosocial determinants play a pivotal role in recovery. In this cross-sectional study, looking at how depression and anxiety relate to quality of life and disability helps us understand the real challenges faced by individuals living with opioid dependence (12).

Against this background, the present study aimed to estimate the prevalence of depression and anxiety among treatment-seeking individuals with opioid dependence, examine their associations with disability and quality of life, and assess socio-demographic and clinical correlates, including factors such as stigma, interpersonal problems, opioid agonist treatment status, and abstinence. Prior literature has shown that comorbid mental disorders are linked to poorer treatment outcomes and psychosocial functioning in opioid use disorder (5, 13), but data from India remain limited. By studying these associations, we sought to generate clinically relevant insights into the psychosocial burden in this population.

## Materials and methods

This paper presents findings from a cross-sectional study conducted at the National Drug Dependence Treatment Centre (NDDTC), Ghaziabad. NDDTC is a speciality Centre of All-India Institute of Medical Sciences (AIIMS), New Delhi an autonomous institute under the Government of India. It provides inpatient and outpatient services to thousands of patients every year. For opioid use disorders, treatment includes opioid substitution therapy, antagonist-based management, and a range of non-pharmacological interventions. Data collection was carried out by MP and PN between May 2023 to January 2025.

Participants included adult patients diagnosed with opioid dependence based on ICD-11 criteria. Inclusion criteria comprised individuals aged 18 years or older, willingness for regular assessments, and availability of a telephone for potential follow-ups. Exclusion criteria were the presence of psychotic disorders or medical and psychiatric conditions interfering with interview-based assessments. The Participants were recruited once they completed the acute withdrawal management phase and were clinically stable thus, not experiencing significant withdrawal or intoxication symptoms, as ensured by the treating psychiatrists. Participants were at different treatment stages; some were on buprenorphine-based opioid agonist therapy during assessment, while others were treatment-naïve or not receiving medication.

Detailed sociodemographic and clinical characteristics were collected for all participants. Sociodemographic variables included age, gender, marital status (currently married or not married), educational level (up to 10th grade or above 10th grade), employment status (currently employed or unemployed), place of residence (urban or rural), family type (living alone, nuclear family, or extended/joint family), having children (yes or no), approximate monthly family income (in INR), and the total number of family members living together. Clinical and opioid-use related characteristics assessed included age at first opioid use (age of first-ever exposure to any opioid), age at regular opioid use (age at which opioid use became a daily or near-daily pattern for at least one month, as reported by the participant), lifetime history of injecting drug use (IDU), sharing injection paraphernalia, history of detention or incarceration due to opioid-related charges, involvement in illegal activities (such as theft) to procure opioids, experience of social stigma related to opioid use (assessed through structured interview questions rather than a standardized scale), presence of interpersonal problems within the family attributed to opioid use, prior history of abstinence from opioids for more than one month, history of admission in treatment facilities (voluntary or coercive), and history of receiving opioid substitution therapy (OST) for more than one month from a designated treatment center.

The baseline assessments involved standardized instruments: Patient Health Questionnaire (PHQ-9) for assessing depression severity, Generalized Anxiety Disorder Scale (GAD-7) for anxiety, WHO Disability Assessment Schedule (WHODAS 2.0) for measuring disability, WHO Quality of Life Bref Scale (WHOQOL Bref) for evaluating quality of life across physical, psychological, social, and environmental domains, and structured evaluations of stigma experiences.

PHQ9: The PHQ-9 is a self-administered depression module of the Patient Health Questionnaire. The questionnaire has 9 questions, and each of the questions is rated from 0 to 3. Scores of 5 and above are indicative of depression (score of 5 to 9 suggestive of mild depression, 10 to 14 of moderate depression, 15 to 19 of moderately severe depression, and more than 19 of severe depression). Internal reliability estimates range from 0.86 to 0.89 using Cronbach’s alpha, and the test-retest reliability is estimated to be 0.84 (14).

GAD7: GAD7 is a seven-item instrument to assess for the symptoms of anxiety. Each of the items is rated on a five-point Likert scale and the total scores range from 0 to 28. Scores of 5, 10, and 15 are taken as the cut-off points for mild, moderate and severe anxiety, respectively. It has been found to be effective for screening three of the common anxiety disorders: panic disorder, social anxiety disorder and post-traumatic stress disorder (15).

WHODAS 2.0: World Health Organization Disability Assessment Schedule version 2.0 (WHODAS 2.0) was used as the gold standard for comparison of IDEAS scores. The 12-item questionnaire was used. All questions have proven psychometric qualities in terms of sensitivity, specificity, reliability, validity and cross population comparability. The simple scoring method was used for domain and total disability scores and then the percentage was calculated (16).

WHOQOL Bref: This is a 26-item questionnaire that is aimed to assess the quality of life. This is self-rated, and a Hindi version is available. Each of the question is rated on a Likert scale of 1 to 5. The questionnaire produces scores on 4 domains: psychological, physical, social and environmental. The two general items on overall satisfaction with life and general health were not included in the analysis. The scale has been used previously in literature pertaining to substance use disorders and has shown good reliability and validity (17).

### Statistical analysis

Data were analyzed using IBM SPSS Statistics (Version 31.0). Descriptive statistics were computed for sociodemographic and clinical variables. Given the non-normal distribution of psychological and functional outcome variables, as confirmed by the Kolmogorov–Smirnov and Shapiro–Wilk tests, non-parametric methods were used. The Mann–Whitney U test and Kruskal–Walli’s test assessed differences in depression, anxiety, quality of life (QOL), and disability scores across categorical variables. Spearman’s rank correlation was used to evaluate associations between continuous variables.

Binary logistic regression was employed to identify independent predictors of moderate-to-severe depression and anxiety, defined by PHQ-9 and GAD-7 cutoffs respectively. Separate logistic regression models were also constructed to examine predictors of low QOL and high disability. Interaction terms were introduced to assess moderation effects between key clinical variables (e.g., stigma × OST, abstinence × interpersonal problems), with significance set at p < 0s.05. All tests were two-tailed, and results were interpreted within the context of clinical relevance and model fit statistics.

## Results

### Sample characteristics

The study population consisted exclusively of male patients (100%) with opioid dependence, with a median age of 28 (24-32) years. The socio-demographics details are given in **Table 1**. Clinically, patients initiated opioid use at a median age of 20 (17-24) years and nearly one-third (29.8%) had a history of injecting drug use, though only 9.4% reported sharing injection equipment. Further clinical details are given in **Table 1**. Heroin was the predominant opioid used (87.1%), with high rates of comorbid tobacco dependence (86.3%). Cannabis use was reported by 29.8%, while alcohol dependence was uncommon (5.9%). The median depression score on the PHQ-9 was 11.00 (interquartile range [IQR] 3.00–19.00). The median anxiety score on the GAD-7 was 8.00 (IQR 2.00–14.00). Disability, as measured by the DAS, had a median score of 6.00 (IQR 1.00–16.00). In terms of quality of life (QOL) domains, the median physical QOL score was 12.00 (IQR 9.71–14.86), psychological QOL was 14.00 (IQR 11.33–16.00), social QOL was 12.00 (IQR 10.67–14.67), and environmental QOL was 15.43 (IQR 12.57–18.29).

**Table 1:** Socio-demographic and clinical variables of the sample (N = 255).

Characteristics	Participants
Age (years)	Median = 28 (24-32)
Gender	Male (100%)
Married	134 (52.5%)
Educated up to 10^th^ grade	191 (74.9%)
Currently employed	217 (85.1%)
Urban residence	205 (80.4%)
Nuclear family	131 (51.4%)
Monthly family income (approx.)	Median: 15,000 INR (10000-22000)
Clinical variables	
Opioid, age at first use	Median age: 20 (17-24) years
Opioid, age at regular use	Median age: 20 (17-24) years
Injecting drug use ever	76 (29.8%)
Sharing of injection or paraphernalia ever	24 (9.4%)
Ever detained or incarcerated due to opioid use or possession	68 (26.7%)
Ever indulged in illegal activities (like theft) for procuring opioids	92 (36.1%)
Currently experience social stigma due to opioid use?	182 (71.4%)
Currently have interpersonal problems with family due to opioid use.	165 (64.7%)
Ever been abstinent to opioids for more than a month	128 (50.2%)
Ever admitted in any facility for opioid dependence	74 (29%)
Ever been forcefully/coercively admitted in any facility for opioid dependence	26 (10.2%)
Ever been on OST for more than a month from a designated Centre	102 (40%)

INR, Indian Rupees; OST, Opioid Substitution Treatment.

To check for Normality of Anxiety, Depression, Disability and Quality of scores, Normality Tests (Shapiro-Wilk & Kolmogorov-Smirnov) were applied. The variables had non-normal distribution (p < 0.001 for all tests). Hence, we have used non-parametric tests for analysis.

### Depression and anxiety categories

Out of 255 participants, 75 (29.4%) had minimal depression, 34 (13.3%) had mild depression, 50 (19.6%) had moderate depression, 40 (15.7%) had moderately severe depression, and 56 (22.0%) had severe depression. With respect to anxiety, 96 (37.6%) had minimal anxiety, 52 (20.4%) had mild anxiety, 59 (23.1%) had moderate anxiety, and 48 (18.8%) had severe anxiety.

As the outcome variables were non-normally distributed, the Mann–Whitney U test was applied for two-group comparisons (marital status, education, employment, and residence), and the Kruskal–Wallis test for family type (three groups). It was seen that employment status showed significant associations with depression, anxiety, all QOL domains, and disability (p < 0.001). Further details are given in **Table 2**.

**Table 2:** Associations between sociodemographic variables and mental health outcomes.

Variable	Groups	Depression	Anxiety	Physical QOL	Psychological QOL	Social QOL	Environmental QOL	Disability
Marital Status	Married vs Non-married	U=7894.000 (p=0.716)	U=7420.000 (p=0.240)	U=7682.500 (p=0.470)	U=7922.000 (p=0.840)	U=7751.500 (p=0.543)	U=7857.500 (p=0.755)	U=8084.000 (p=0.969)
Education	Lower vs Higher	U=5595.000 (p=0.310)	U=5601.500 (p=0.315)	U=5751.000 (p=0.479)	U=5873.500 (p=0.684)	U=5991.500 (p=0.812)	U=5851.000 (p=0.652)	U=6017.500 (p=0.852)
Employment	Unemployed vs Employed	U=2368.500 (p<0.001)*	U=2244.000 (p<0.001)*	U=2101.000 (p<0.001)*	U=2647.000 (p<0.001)*	U=2476.500 (p<0.001)*	U=2113.500 (p<0.001)*	U=2450.500 (p<0.001)*
Residence	Rural vs Urban	U=4491.000 (p=0.174)	U=4994.500 (p=0.779)	U=4432.000 (p=0.138)	U=4737.000 (p=0.536)	U=4954.500 (p=0.713)	U=4715.500 (p=0.506)	U=5065.000 (p=0.897)

*Significant at p < 0.05. QOL, Quality of Life. Statistically significant associations are highlighted in bold.

The Kruskal-Wallis test revealed a statistically significant difference in social quality of life across family types (H = 7.125, p=0.028), while no significant differences were found for depression (H = 3.058, p=0.217), anxiety (H = 2.938, p=0.230), disability (H = 0.514, p=0.773), physical QOL (H = 1.795, p=0.408), psychological QOL (H = 2.657, p=0.265), or environmental QOL (H = 4.746, p=0.093), suggesting that family type primarily influences social functioning rather than other mental health or quality of life domains in this sample.

### Correlation of depression and anxiety with related psychosocial measures

Spearman correlation analysis revealed a strong positive association between depression (PHQ-9) and anxiety (GAD-7) scores (r = 0.903, p < 0.001), indicating a significant co-occurrence of these mental health symptoms. Both depression and anxiety were also positively correlated with overall disability levels (WHODAS) (r = 0.797 and r = 0.791, respectively; both p < 0.001). Importantly, both depression and anxiety scores demonstrated strong negative correlations with all four domains of quality of life. Depression was most strongly negatively correlated with physical quality of life (r = -0.853, p < 0.001), followed by environmental (r = -0.758), psychological (r = -0.741), and social domains (r = -0.706). Similar patterns were seen for anxiety scores, with significant negative correlations observed across physical (r = -0.831), environmental (r = -0.723), psychological (r = -0.727), and social domains (r = -0.680), all p-values being < 0.001. These findings suggest that elevated symptoms of depression and anxiety are robustly associated with lower quality of life across all measured domains.

There was no statistically significant association between depression or anxiety scores and variables such as injection drug use, history of incarceration, or forced admission (p > 0.05 for all). However, participants reporting experienced stigma, lack of abstinence in the past month, reporting interpersonal problems due to opioid use and not receiving opioid substitution therapy (OST) showed significantly higher levels of both depression and anxiety (all p-values < 0.001). In terms of continuous variables, higher per capita income was negatively correlated with both depression (r = -0.327, p < 0.001) and anxiety scores (r = -0.307, p < 0.001), suggesting a protective effect. Additionally, earlier age at regular opioid use was weakly but significantly associated with higher anxiety levels (r = -0.132, p = 0.036), while age at first opioid use did not show significant correlation with either outcome (**Supplementary Table 1**).

Quality of life and disability outcomes were significantly influenced by multiple clinical factors. Stigma, lack of abstinence in the past month, interpersonal problems, and not receiving opioid substitution therapy were all significantly associated with lower scores across physical, psychological, social, and environmental QOL domains (all p <.001). These same variables also showed significant associations with higher disability scores. Spearman correlations revealed that per capita income was positively correlated with all QOL domains (r ranging from.212 to.427, all p <.001), and negatively correlated with disability (r = -0.369, p <.001), indicating a protective effect. Age at regular opioid use also showed mild but significant positive associations with psychological and environmental QOL.

In contrast, injection drug use, incarceration history, forced admissions, and substance use variables (tobacco, alcohol, cannabis) were not consistently associated with QOL or disability outcomes (**Supplementary Table 2**).

### Logistic regression results: predictors of moderate-to-severe depression (PHQ-9 ≥10)

In line with prior literature, PHQ-9 scores were dichotomized into minimal–mild (<10) versus moderate–to–severe (≥10). A cut-off of 10 on the PHQ-9 has been shown to provide high sensitivity and specificity for major depression (14) and has been consistently validated as an optimal threshold in large-scale meta-analyses (18). This grouping enabled us to differentiate participants with subthreshold symptoms from those with clinically significant depressive symptoms likely to impair functioning and require intervention. A binary logistic regression was conducted to examine predictors of moderate-to-severe depression among patients.

The model was statistically significant (χ²(5) = 95.364, p <.001) and explained 41.9% of the variance in depression (Nagelkerke R² = .419). The Hosmer and Lemeshow test indicated good model fit (χ² = 7.649, p = .469). Overall classification accuracy was 77.3%.

Significant predictors of depression included: stigma (OR = 2.95, 95% CI: 1.37–6.38, p = .006), absence of opioid substitution therapy (OR = 0.30, 95% CI: 0.15–0.60, p <.001), and presence of interpersonal problems (OR = 4.21, 95% CI: 2.03–8.77, p <.001). Abstinence and per capita income were not statistically significant predictors in the multivariate model (**Table 3**).

**Table 3:** Multivariable logistic regression predicting depression (PHQ-9 ≥10).

Variable	B	S.E.	Wald	P-value	Exp (B) (OR)	95% CI lower	95% CI upper
Stigma (Yes vs No)	1.082	0.393	7.577	0.006*	2.952	1.366	6.38
Abstinence (Yes vs No)	-0.488	0.335	2.124	0.145	0.614	0.319	1.183
OST (Yes vs No)	-1.207	0.355	11.536	0.001*	0.299	0.149	0.6
Interpersonal Problems (Yes vs No)	1.438	0.374	14.796	<0.001*	4.213	2.025	8.768
Per Capita Income	0.0	0.0	0.002	0.965	1.0	1.0	1.0

Independent variables initially considered for the model included age, marital status, education, employment, residence, per capita income, age at first and regular opioid use, injecting drug use, incarceration, stigma, abstinence, opioid substitution therapy (OST) status, interpersonal problems, and comorbid substance use (tobacco, alcohol, cannabis). Based on univariate analyses and clinical relevance, the final model retained stigma, interpersonal problems, OST status, abstinence, and per capita income. Statistically significant associations are highlighted in bold.

A binary logistic regression was conducted to examine the interaction between stigma and opioid substitution therapy (OST) in predicting moderate-to-severe depression (PHQ-9 ≥10). The model was statistically significant, χ²(3) = 82.431, p <.001, with a Nagelkerke R² of.371, indicating that 37.1% of the variance in depression was explained. The Hosmer–Lemeshow test showed good model fit (χ² = 0.000, p = 1.000), and the overall classification accuracy was 72.2%.

The interaction between stigma and OST was significant (OR = 6.26, 95% CI: 1.12–35.04, p = .037), indicating that the protective effect of OST is attenuated in individuals who report stigma. In other words, OST is more effective in reducing depression when stigma is low (**Supplementary Table 3**).

### Logistic regression results: predictors of moderate-to-severe anxiety (GAD-7 ≥10)

A binary logistic regression was performed to identify predictors of moderate-to-severe anxiety among patients. The model was statistically significant (χ²(5) = 65.953, p <.001), with a Nagelkerke R² of.307, indicating that approximately 30.7% of the variance in anxiety levels was explained by the model. The Hosmer and Lemeshow test showed a good model fit (χ² = 5.789, p = .671). The overall classification accuracy of the model was 70.6%.

Significant predictors of anxiety included: interpersonal problems (OR = 4.98, 95% CI: 2.24–11.11, p <.001) and abstinence in the past month, which was protective (OR = 0.45, 95% CI: 0.25–0.82, p = .009). Stigma, OST status, and per capita income were not statistically significant predictors in the model (**Table 4**).

**Table 4:** Multivariable logistic regression predicting anxiety (GAD-7 ≥10).

Variable	B	S.E.	Wald	P-value	Exp (B) (OR)	95% CI lower	95% CI upper
Stigma (Yes vs No)	0.469	0.411	1.301	0.254	1.598	0.714	3.573
Abstinence (Yes vs No)	-0.797	0.307	6.741	0.009*	0.451	0.247	0.823
OST (Yes vs No)	-0.179	0.348	0.263	0.608	0.836	0.423	1.655
Interpersonal Problems (Yes vs No)	1.606	0.409	15.416	<0.001*	4.984	2.235	11.112
Per Capita Income	0.0	0.0	2.198	0.138	1.0	1.0	1.0

Independent variables initially considered included age, marital status, education, employment, residence, per capita income, age at first and regular opioid use, injecting drug use, incarceration, stigma, abstinence, OST status, interpersonal problems, and comorbid substance use. In the final model, interpersonal problems and abstinence emerged as significant predictors. Statistically significant associations are highlighted in bold.

A binary logistic regression was conducted to examine whether the association between abstinence in the past month and moderate-to-severe anxiety (GAD-7 ≥10) was moderated by interpersonal problems. The overall model was statistically significant (χ²(3) = 60.68, p <.001), with a Nagelkerke R² of 0.286, indicating that the model explained approximately 28.6% of the variance in anxiety. The Hosmer-Lemeshow test suggested a good model fit (p = 1.000). The overall classification accuracy was 69.7%.

Main effects were observed for abstinence (OR = 0.27, 95% CI: 0.08–0.94, p = .039), indicating a protective effect, and interpersonal problems (OR = 5.65, 95% CI: 2.18–14.62, p <.001), suggesting significantly increased odds of anxiety. However, the interaction between abstinence and interpersonal problems was not statistically significant (p = .455), suggesting that the protective effect of abstinence did not significantly differ by the presence or absence of interpersonal problems (**Supplementary Table 4**).

## Discussion

The study sample comprised exclusively of men with a median age of 28 years which reflected the demographic pattern of treatment-seeking opioid users in India. Most participants were urban residents, educated up to the tenth grade, and currently employed. Heroin was the predominant opioid (87%), and nearly one-third had a history of injecting drug use. A large majority reported stigma (71%) and interpersonal problems within the family (65%), and only 40% had ever received opioid substitution therapy. These demographic and clinical patterns underscore the young, socially burdened, and predominantly working-age male profile of Indian opioid-dependent populations, similar to earlier national surveys.^2^

Our study shows that people with opioid dependence carry a heavy burden of mental health problems. More than half (57%) had moderate to severe depression, and over two in five (42%) had moderate to severe anxiety. These two conditions were also very closely correlated with each other (r = 0.90). The rates we observed are higher than the pooled estimates reported in the largest global review to date, which found current depression in 36% and anxiety in 29% of people with opioid use disorder. Importantly, that review highlighted wide variability across studies, with prevalence ranging from about 15% to over 60% depending on the population, instruments used, and recruitment setting (5). Several factors may explain why our estimates are on the higher end. First, our sample comprised treatment-seeking individuals, likely experiencing greater psychosocial stressors. Secondly, we used symptom screening scales (PHQ-9, GAD-7) rather than diagnostic interviews; these tools may identify more cases and some items (e.g. sleep, fatigue, concentration) may overlap with opioid withdrawal or chronic use effects, potentially inflating scores. Hence, these considerations suggest that while our estimates are higher, they are consistent with the higher range of variation reported globally.

The high overlap between depression and anxiety appears to be more than a statistical finding with various clinical implications. Previous studies show that people with opioid dependence who also have depression are more likely to experience overdoses, suicidal behavior, other substance use disorders, and greater use of health services (8, 19). Previous studies from South Asia and the Middle East echoes our findings, reporting similarly high levels of mood and anxiety symptoms in opioid-dependent populations (6, 7). Anxiety disorders, in particular, appear especially common among people who inject drugs (PWID) (20). Taken together, the results from this study suggest that the individuals in our study represent a highly vulnerable clinical group, with mental health needs that are at least as pressing as their substance use treatment needs.

In our study, both depression and anxiety were closely related with greater disability and poorer quality of life across physical, psychological, social, and environmental areas. The strongest link was between depression and physical quality of life (r = −0.85). This finding is consistent with previous studies showing that psychological distress, more than the severity of drug use itself, is the main factor driving poorer quality of life in people with opioid dependence (12). Previous studies have also reported that individuals with opioid use disorder experience significant declines in both physical and mental health-related quality of life compared with the general population (9). Similarly, meta-analyses across substance use disorders highlight that the presence of co-occurring psychiatric conditions predicts worse outcomes across physical, mental, social, and environmental domains of functioning (21). In the Indian context, stigma adds another layer, with evidence showing that people with opioid dependence face widespread quality-of-life impairments due to discrimination and social exclusion (10). Thus, our findings underline that addressing psychiatric symptoms is not just about improving mental health but it is central to enhancing overall functioning and quality of life in those with opioid dependence.

In the study, employment status emerged as a strong factor associated with psychiatric symptoms, disability, and quality of life, while other demographic variables showed little effect. This finding is in line with broader population data showing that low socioeconomic status including unemployment and low income is associated with long-term opioid use and poorer outcomes (13). Among people receiving treatment, financial situation has consistently emerged as one of the few predictors of improvement in quality of life over time (22). Similarly, studies of long-term opioid agonist therapy (OAT) patients show that health-related quality of life remains lower than population norms, especially at older ages (23). In this context, the protective effect of higher per-capita income in our study is unsurprising and highlights the importance of incorporating income and employment support into recovery-oriented care for opioid dependence.

In our study, stigma, lack of abstinence, interpersonal difficulties, and not being on opioid substitution therapy (OST) were associated with more severe psychiatric symptoms, higher disability, and poorer quality of life. Also, what is seen from previous studies is that both internalized and external stigma are strongly tied to depression, anxiety, low self-esteem, social exclusion, and worse quality of life among people with opioid use disorder (10, 11). Stigma within healthcare settings, in particular, has been reported as a barrier that discourages disclosure and help-seeking among OST recipients (24). Similarly, interpersonal adversities such as rejection and conflict are known to heighten anxiety in opioid-dependent groups (25). The role of ongoing opioid use is also important, longer or heavier exposure is associated with worsening mood and anxiety symptoms over time (8).

In our adjusted models, depression (PHQ-9 ≥ 10) was most strongly predicted by stigma (OR ≈ 3) and interpersonal difficulties (OR ≈ 4), while being on OST showed a protective effect. Notably, we found a stigma × OST interaction, suggesting that stigma diminishes the protective effect of OST on depressive symptoms. Although few studies have directly studied this interaction, the finding makes conceptual sense. Stigma is a well-documented barrier in opioid dependence care as it hinders treatment entry, weakens engagement and retention, and reduces confidence in treatment effectiveness (26, 27). As discussed earlier stigma in any form is strongly linked with depression, anxiety, low self-esteem, social exclusion, and poorer quality of life (10, 11). Within healthcare settings also stigma is known to deter disclosure and help-seeking among OST recipients (24). This implies that medication alone may not be enough if stigma is not addressed, its shadow can blunt the full benefit of pharmacotherapy.

For anxiety (GAD-7 ≥ 10), interpersonal problems were the strongest predictor (OR ≈ 5), while past-month abstinence was protective (OR ≈ 0.45). The non-significant abstinence × interpersonal-problems interaction suggests these influences act additively rather than synergistically. This is consistent with prior evidence linking interpersonal adversity to heightened anxiety in opioid use disorder (25). In our sample, being on opioid agonist treatment (OAT) was also associated with lower odds of anxiety, echoing international evidence that reducing substance use or engaging in OAT improves overall wellbeing over time (28, 29).

Our findings have several important implications for clinical practice and service delivery. First, mental health screening should become a routine part of care for people with opioid use disorder. Simple tools such as the PHQ-9 and GAD-7 can help identify depression and anxiety early, which is crucial given how strongly these symptoms affect day-to-day functioning and quality of life (5, 9). Just as important is the need to tackle stigma. Training staff to use respectful, non-stigmatizing language, creating supportive policies, and involving peers in care can make treatment more welcoming and strengthen the benefits of opioid substitution therapy, as our results on the stigma × OST interaction suggest (24, 26). Interpersonal relationships also equally crucial. Therapy approaches that focus on relationships whether through family involvement, social support, or structured interventions like interpersonal therapy may reduce the impact of interpersonal difficulties on depression and anxiety (25, 30). Beyond clinical care, socioeconomic support should be seen as a core part of recovery. Help with employment, vocational training, or financial assistance not only improves quality of life but also reduces the risk of ongoing opioid use (13, 22). Finally, services must continue to prioritize sustained engagement with OST and support for abstinence, as both are strongly linked with better mental health and improved functioning (12, 28, 29).

This study has a number of strengths. We were able to assess psychiatric symptoms, disability, and multiple domains of quality of life at the same time, using well-validated instruments. We also went beyond simple associations by applying multivariable models and testing theoretically informed interactions, such as stigma × OST and abstinence × interpersonal problems. However, our study also had several limitations. The cross-sectional design of the study prevents us from drawing conclusions about causality, and the single-center setting limits the representativeness of the findings. Our sample was recruited purposively from a tertiary-care facility, which may have led to selection bias by preferentially including individuals with more severe illness or greater treatment needs. Furthermore, we relied on self-report screening tools rather than structured diagnostic interviews, which may have inflated prevalence estimates, particularly as some items overlap with symptoms of opioid use or withdrawal. Finally, the generalizability of our findings is likely to vary depending on local service structures and prevailing stigma climates, which differ across settings.

## Conclusion

In conclusion, our findings highlight the substantial burden of depression and anxiety among people with opioid dependence and their associations with disability and quality of life. These results highlight the importance of routine screening and integrated psychosocial support alongside opioid agonist treatment in clinical settings.

## Future directions

Future research should add on these findings through longitudinal and multi-center studies that can clarify causal pathways, assess variation across service contexts, and evaluate the effectiveness of targeted interventions. In particular, studies employing diagnostic interviews, diverse recruitment strategies, and follow-up designs will be crucial for advancing our understanding of how mental health and opioid dependence interact over time and how best to address this dual burden.

## Data Availability

The raw data supporting the conclusions of this article will be made available by the authors, without undue reservation.
