# Supplementary material for: Psychosocial correlates of depression and anxiety among treatment-seeking individuals with opioid dependence: a cross-sectional study
Source: Front Psychiatry. 2025 Nov 5;16:1708666. doi: 10.3389/fpsyt.2025.1708666 (PMC12626948; doi:10.3389/fpsyt.2025.1708666)
Supplement: Supplementary file 1 [file Table1.pdf]

Supplementary Tables

Supplementary Table 1: Associations of Depression and Anxiety with Clinical Parameters

Clinical Parameters	Depression (PHQ-9)	Anxiety (GAD-7)
Injection Drug Use (IDU)	U = 6167.0, p = .237	U = 6004.0, p = .136
Incarceration History	U = 5885.5, p = .363	U = 5994.5, p = .483
**Experienced Stigma**	U = 2910.5, **p < .001***	U = 3141.0, **p < .001***
**Abstinence in Past Month**	U = 4709.0, **p < .001***	U = 4701.0, **p < .001***
**On Opioid Substitution Therapy**	U = 3532.0**, p < .001***	U = 4062.5**, p < .001***
Forced Admission	U = 2972.0, p = .989	U = 2868.0, p = .758
**Interpersonal problems due to opioid use**	U = 2331.0, **p < .001***	U = 2716.0, **p < .001***
Tobacco Dependence	U = 3663.0, p = .521	U = 3629.0, p = .468
Alcohol Dependence	U = 1642.5, p = .569	U = 1776.5, p = .932
Cannabis Dependence	U = 6111.0, p = .208	U = 6191.0, p = .299
Age at First Opioid Use	r = -0.060, p = .342	r = -0.104, p = .097
**Age at Regular Use**	r = -0.090, p = .151	r = -0.132, **p = .036**
**Per Capita Income**	r = -0.327, **p < .001***	r = -0.307, **p < .001***

Significant associations are highlighted, * p < 0.05

Supplementary Table 2: Associations of Clinical Parameters with Quality of Life and Disability

**Clinical Parameters**	**Physical QOL**	**Psychological QOL**	**Social QOL**	**Environmental QOL**	**Disability (DAS)**
Injection Drug Use	U = 6306.0, p = .357	U = 6097.0, p = .248	U = 5729.0, **p = .045***	U = 6393.0, p = .549	U = 6368.5, p = .418
Incarceration History	U = 6087.5, p = .603	U = 6213.5, p = .921	U = 5877.5, p = .353	U = 6124.5, p = .786	U = 5834.0, p = .312
**Stigma**	U = 3106.5, **p < .001***	U = 3066.0, **p < .001***	U = 3448.0, **p < .001***	U = 3274.0, **p < .001***	U = 3043.0, **p < .001***
**Abstinence** (Past Month)	U = 4514.0, **p < .001***	U = 5669.5, **p < .001***	U = 5765.5, **p < .001***	U = 4765.0, **p < .001***	U = 4484.0, **p < .001***
Opioid Substitution Therapy	U = 2737.5, p = .501	U = 2768.0, p = .580	U = 2827.0, p = .672	U = 2588.0, p = .289	U = 4422.5, **p < .001***
Forced Admission	U = 2737.5, p = .501	U = 2768.0, p = .580	U = 2827.0, p = .672	U = 2588.0, p = .289	U = 2849.0, p = .718
**Interpersonal Problems**	U = 2358.0, **p < .001***	U = 3162.5, **p < .001***	U = 2454.0, **p < .001***	U = 2247.5, **p < .001***	U = 2657.5, **p < .001***
Tobacco Dependence	U = 3862.5, p = .880	U = 3101.0, **p = .047***	U = 2958.0, **p = .017***	U = 3714.5, p = .637	U = 3640.5, p = .485
Alcohol Dependence	U = 1731.5, p = .804	U = 1520.5, p = .550	U = 1302.5, p = .071	U = 1484.5, p = .464	U = 1750.0, p = .856
Cannabis Dependence	U = 6268.5, p = .426	U = 6296.5, p = .181	U = 6085.0, **p = .029***	U = 6272.0, p = .383	U = 6398.5, p = .320
Age at First Opioid Use	r = .101, p = .109	r = .127, **p = .044***	r = .097, p = .122	r = .118, p = .059	r = -0.058, p = .360 (Spearman)
Age at Regular Opioid Use	r = .116, p = .064	r = .132, **p = .035**	r = .111, p = .078	r = .118, p = .060	r = -0.074, p = .240 (Spearman)
Per Capita Income	r = .399, **p < .001***	r = .212, **p < .001***	r = .310, **p < .001***	r = .427, **p < .001***	r = -0.369, **p < .001*** (Spearman)

Significant associations are highlighted, * p < 0.05

Supplementary Table 3: Logistic Regression with Interaction Term (Stigma × OST)

Predictor	B	SE	Wald	p-value	OR (Exp B)	95% CI Lower	95% CI Upper
**Stigma** (Yes vs No)	1.162	0.425	7.474	**0.006***	3.195	1.389	7.349
**OST** (Yes vs No)	-3.19	0.809	15.529	**<.001***	0.041	0.008	0.201
**Stigma × OST**	1.834	0.879	4.356	**0.037***	6.259	1.118	35.037
Constant	0.194	0.361	0.289	0.591	1.214		

The model included stigma, OST status, and their interaction term, with adjustment for abstinence, interpersonal problems, and per capita income. Significant associations are highlighted, * $p < 0.05$

Supplementary Table 4: Logistic Regression Predicting Anxiety (GAD-7 ≥10) with Abstinence × Interpersonal Interaction

Variable	B	S.E.	p-value	Exp(B) (OR)	95% CI Lower	95% CI Upper
**Abstinence** (Yes vs No)	-1.317	0.639	**0.039***	0.268	0.077	0.937
**Interpersonal Problems** (Yes vs No)	1.731	0.485	**<.001***	5.647	2.181	14.619
Abstinence × Interpersonal Problems	0.535	0.717	0.455	1.708	0.419	6.956

The model included abstinence, interpersonal problems, and their interaction term, with adjustment for stigma, OST status, and per capita income. Significant associations are highlighted, * $p < 0.05$
